# Laws of macroevolutionary expansion

**DOI:** 10.1073/pnas.2314694121

**Published:** 2024-08-06

**Authors:** Indrė Žliobaitė

**Affiliations:** ^a^Department of Computer Science, University of Helsinki, Helsinki 00014, Finland; ^b^Department of Geosciences and Geography, University of Helsinki, Helsinki 00014, Finland; ^c^Finnish Museum of Natural History (LUOMUS), Helsinki 00014, Finland

**Keywords:** Law of constant extinction, the Red Queen’s hypothesis, macroevolution, expansive energy, species ranges

## Abstract

Van Valen’s law of constant extinction postulates that in comparable ecological contexts, the probability for a taxon to survive to the next time interval is independent of how long it has already existed. The law implies that species do not age, that is, the survival probability from one time step to the next does not decrease with species’ age. While the original law describes survivorship over time, here we show that there is a counterpart to the law describing species dynamics in space. Indeed, the probability for a taxon to expand a step further in its range size does not depend on how large of a range the taxon already occupies. We show that such patterns of stochastically constant expansion are common in the global mammalian fossil record throughout the Cenozoic and are consistent with the mammalian species distribution in the present-day world. Intriguing is that the law in space holds best for the linear dimension of the range rather than for the range area. We interpret this as suggesting that species primarily expand via the frontier of their range; this process is known as leading-edge expansion in ecology. And while the difficulty of expanding in linear steps remains constant, the area gains diminish. In line with evolutionary theory collectively known as the Red Queen’s hypothesis, this suggests that the effectiveness of expansion saturates, even if species longevity does not. When interpreted in reverse, this explains from the macroevolutionary perspective why larger species ranges are associated with lower extinction risks.

## Significance

We analyze macroevolutionary dynamics of mammalian species in time and space. We find that the chance to expand by one linear step in space does not depend on how large of a range a species already occupies. Our results suggest that macroevolutionary expansion primarily happens through the perimeter of the species’ range. The results contribute to evolutionary theory by offering insights into how macroevolutionary expansion and extinction relate to range changes. Our empirical analysis linking the global mammalian fossil record of the Cenozoic with the distribution of mammalian species in the present-day world can help better understand extinction dynamics in the present-day ecosystems.

The life history and arguably success of each species can be described by how long the species existed and how abundant or widely spread it was. While we know that considered in the same ecological contexts species’ survival in time is memoryless (1–3), implying that the probability of extinction of a species in the next time interval does not depend on how long it has already existed, we know much less of macroevolutionary dynamics of species expansion and decline in space.

We know that unimodal patterns of rise and decline in abundance, occupancy, or range are very common in the fossil record of terrestrial mammals (4–7), marine invertebrates (8–10), marine microorganisms (11), as well as recent clades of animals and plants (12), even if there are exceptions (6). Whether unimodal or multimodal, each taxon at some point reaches a peak in its geographic range size (13, 14) and its range expansion towards the peak could be rapid or slow (15, 16). Thus, even if the trajectory of a species expansion and decline is not unimodal or not symmetric in time, we can characterize each species by how long it existed and how widely it was spread throughout its existence. From there we can ask, even if taxa do not age (the survival probability from one time step to the next does not decrease with age) (1), how does the expansion probability of a taxon depend on its current range size? In an operational sense, this question translates to whether the probability of finding a taxon with an even larger range size increases with range size.

This suggests a hypothetical possibility for species’ expansion to follow the power law, where the probability of the next outcome to take a particular value is proportional to the number of outcomes already having that value. The power law is typically used to model allometric scaling of longevity (17) or home ranges (18) of individuals with their body mass, or species diversity, such as in the species-area relationships (19). The main alternative is a possibility for expansion in space to follow memoryless decay, similarly to survivorship of taxa in time as in Van Valen’s law of constant extinction (1, 3), especially since the underlying Red Queen’s process primarily characterizes expansion (7). The catch is that taxa can expand rapidly or slowly (15, 20) and the peak range size of a species in the mammalian fossil record at most mildly correlates with species longevity (plotted in supplement). We ask, what macroevolutionary laws, then, govern expansion in space?

## The Law of Constant Extinction

**Fig. 1.**
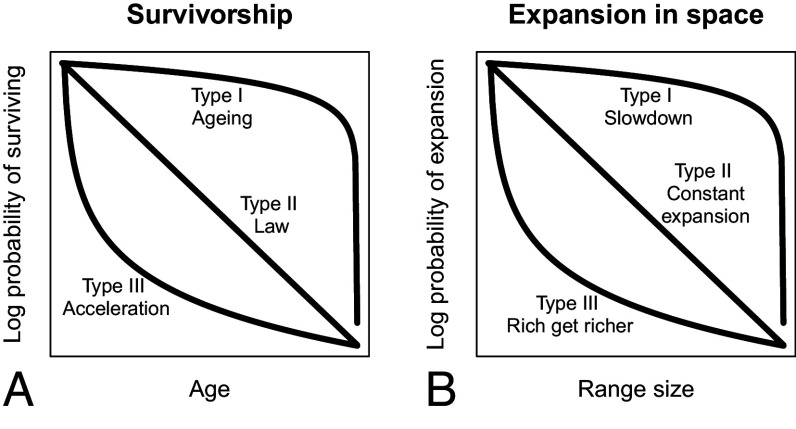
An illustration of the main types of survivorship curves. (*A*) Survivorship curves for individuals or taxa, the curves plot the proportion of individuals or taxa alive at a particular age. (*B*) Expansion curves for range sizes of taxa, the curves plot the proportion of taxa that have ever reached a larger range size than the thresholds given on the horizontal axis.

Van Valen’s law of constant extinction (1) postulates that in the same ecological contexts (21), the probability of extinction of a taxon during the next time interval does not depend on its age. Van Valen discovered these patterns when analyzing survivorship of taxonomic lineages in the fossil record in a way that has been common for demographic analysis of individuals (22), the approach is known as analysis of survivorship curves in ecology. When the proportion of individuals alive at a specific age is plotted against that age on a semilog plot, the shape of the curve can be used for reasoning about the probabilities of surviving from one age group to the next. This relationship can be of three types, as illustrated in Fig. 1*A*. In Type I relationship, the probability of dying in the next time step accelerates with the individual’s age. This reasoning is inferred from the slope of the survivorship curve being shallower at younger ages and steeper at older ages. This implies that the mortality rate is higher at older ages. In Type III relationship, the probability of dying decreases with age. This reasoning is inferred from the slope of the survivorship curve being steeper at younger ages, which implies that the mortality rate is higher for younger individuals. Type II relationship, which plots as a straight line on a semilog plot, implies that the probability of dying during the next time interval is the same for individuals of all ages.

While mortality of individuals in a population rarely follows Type II relationship (23), Van Valen discovered that longevity of taxonomic groups in the terrestrial and marine fossil record commonly do (1), implying that the probability of going extinct in the next time step in the same ecological contexts does not depend on how long a taxon has already existed. Type II survivorship can be modeled as an exponential decay (3), commonly used to describe the behavior of systems with replaceable parts, such as times to mechanical breakdowns, durations of phone calls, or decay of unstable isotopes. Consistently with the notion of replaceable parts, which in the macroevolutionary realm are individuals belonging to the same taxon, Van Valen’s Red Queen’s hypothesis (1, 7) explains the patterns of evolutionary decay arising from a never-ending zero-sum game (24) where “no species can ever win and new adversaries grinningly replace the losers” (1). A possibility of a new lineage arising from within the system makes the process memoryless (20). No matter how well an individual is adapted, its close relative or even its own local descendant (6) can be all the same but slightly better adapted for the current circumstances, and thus at any time give a rise to a new lineage without a need to start accumulating traits from scratch.

Our analysis first revisits the original law of constant extinction, which concerns survivorship of species in the mammalian fossil record. Previously the law has been tested at the family (1, 25) or genus level (7, 26, 27). To the best of our knowledge, the law has not yet been systematically tested at the species level for mammals. While the genus or higher taxonomic levels add robustness to analyses (28), species is the ultimate unit of evolutionary competition. Our computational treatment gives sufficient resolution to analyze survivorship patterns at the species level.

**Fig. 2.**
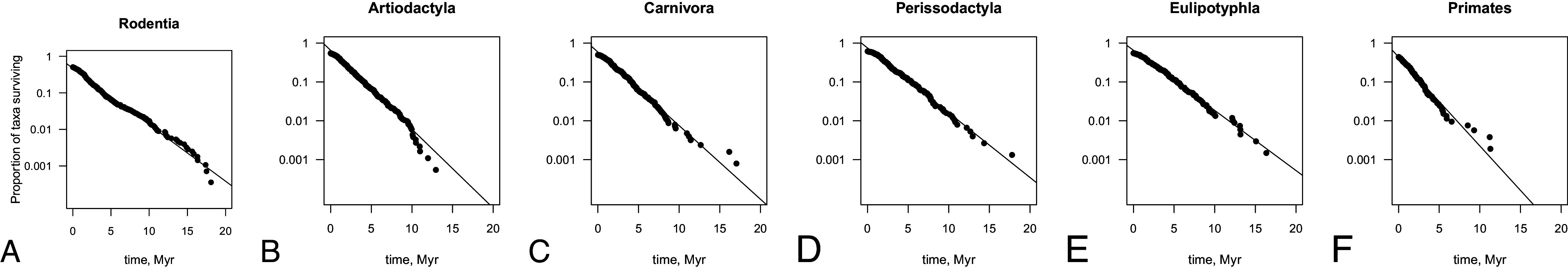
Species survivorship curves for six largest orders of mammals in the global fossil record throughout the Cenozoic. (*A*) Rodentia, (*B*) Artiodactyla, (*C*) Carnivora, (*D*) Perissodactyla, (*E*) Eulipotyphla, (*F*) Primates. This analysis is at the species level. Axes are the same in all plots for comparability. Plots for all the mammalian orders are given in the online repository.

The Fig. 2 plots the species survivorship curves for six largest orders of mammals from the NOW database of fossil mammals (29). The relationships are convincingly linear suggesting that the law of constant extinction holds. We can also see that the slopes across orders are quite similar suggesting comparable average extinction rates across these major orders.

The off-the-slope tails at the longest durations are likely due to noise on identification of species as well as uncertainties in age estimations in the source data. For completeness of the treatment, we did not manually remove any presumed outliers from the data beyond the regular ongoing curatorial process of the NOW database, in which the author of this analysis is actively involved.

## Patterns of Expansion

We can analyze species expansion in space in a similar way to their survivorship in time. Given the range that a species occupies, we can ask what the probability is of ever occupying a larger range. We can formalize this analysis of range expansion in a similar way to species survivorship, where points on the horizontal axis (Fig. 1*A*) represent species of a particular age, where some species go extinct at that age, others continue to exist. We can construct expansion in space curves in a similar way, illustrated in Fig. 1*B*, where points on the horizontal axis represent particular range sizes. These expansion curves depict the proportion of taxa that have a larger range than the threshold on the horizontal axis. This is analogous to being alive in the survivorship curves. As we move horizontally along the expansion curve, some species reach their peak range, others continue expanding. The timing of expansion is not part of this formulation; the question is only whether they will eventually have a larger range.

Our analytical task is to investigate whether the probability of expansion in space depends on how large of a range the taxon already occupies. Similarly to survivorship we infer the probability of expansion from the slope of the curve. If the curve is linear in the semilog plot then the probability of range expansion by one step in space does not depend on how large range a taxon already occupies. Analytically we can ask whether the expansion curves observed in the fossil record closer follow Type I, Type II, or Type III pattern. If the best fit is Type I pattern, we can conclude that expansion in space saturates, taxa occupying large ranges are less likely to expand to even larger ranges. While eventually saturation happens anyway, as land masses are discrete and finite in space, the type of curve indicates what happens far before the limit of land masses is reached. If the best fit is Type III pattern, we can conclude that the “rich get richer” pattern holds; taxa that have large ranges are more likely to expand further. Finally, if Type II pattern gives the best fit, we can conclude that the expansion process in space is memoryless similarly to survivorship of taxa in time, following the Red Queen’s theory.

The process in space can be memoryless in relation to taxon’s range area, or, alternatively, it can be memoryless in relation to the linear dimension of taxon’s range, which can be thought of as the width or the diameter of the range. These alternative best fits would carry different macroevolutionary interpretations. Thus, for the ranges, we test whether Type I, Type II, or Type III is better supported in relation to range width (in km) or range area (in km^2^).

**Fig. 3.**
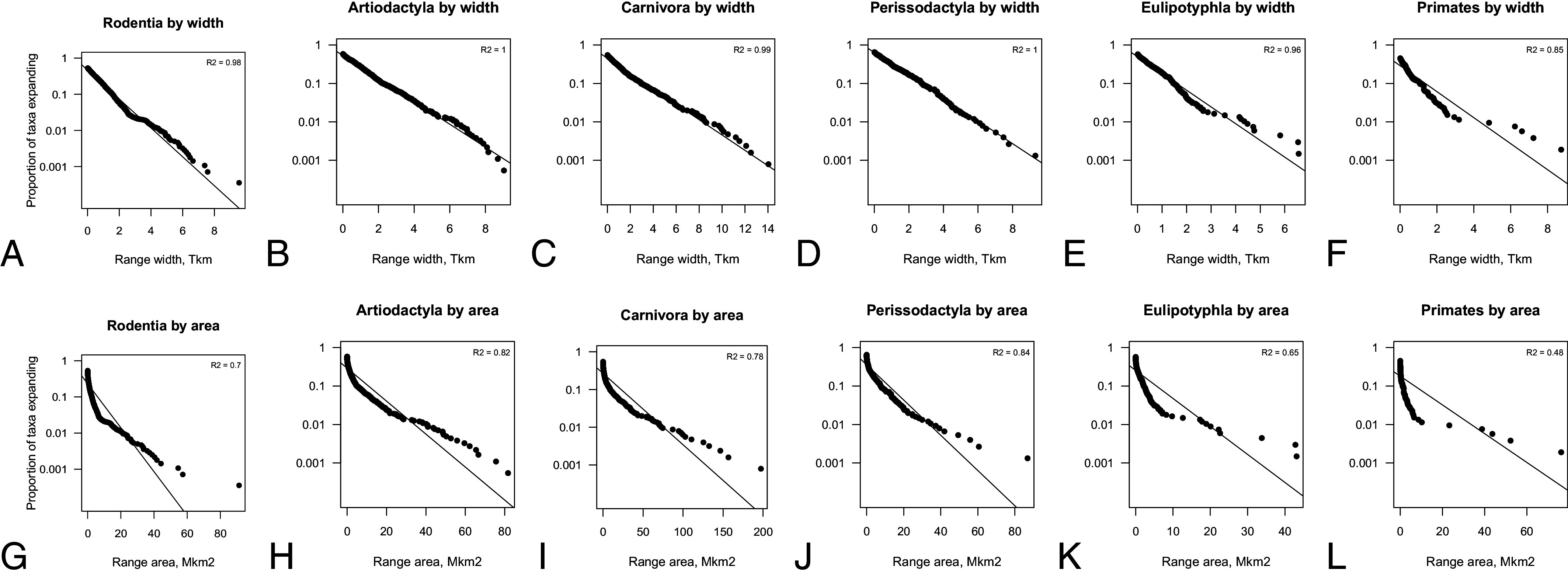
Species maximum range expansion curves for six largest orders of mammals in the fossil record, by range width (*A*–*F*) and by range area (*G–L*). This analysis is at the species level. The horizontal axis differs from plot to plot. The vertical axis is the same in all plots. Plots for all the mammalian orders are given in the online repository.

Fig. 3 shows patterns of the maximum range expansion across six most abundant mammalian orders in the fossil record. The fits by widths of the ranges in Fig. 3 *A–F* generally appear to be linear, and the fits in Fig. 3 *A–F* are consistently tighter than the fits by areas in Fig. 3 *G–L*. Table 1 summarizes the outcomes for all the orders in the mammalian fossil record that have at least 10 data points for fitting the curves. We report survivorship and expansion curves in the fossil record as well as curves for the mammalian occurrence data at present-day. Visualizations and fit statistics for each order separately are given in an online repository (https://github.com/zliobaite/expansion). The table reports the results at the species as well as at the genus level.

**Table 1.** The patterns of taxon survivorship and expansion in the global fossil record throughout the Cenozoic as well as the global species distribution at the present-day

		Van Valen’s law of constant extinction		Patterns of expansion			
		Survivorship of taxa, fossils		Ranges of taxa, fossils		Ranges of taxa, present-day	
	No. of occurrences at the genus level in the fossil data	by genera	by species	by genera	by species	by species	by genera
Rodentia	20,426	LAW	LAW	LAW	LAW	LAW	LAW
Artiodactyla	15,410	LAW	LAW	LAW	LAW	LAW	LAW
Carnivora	9,712	LAW	LAW	LAW	LAW	LAW	LAW
Perissodactyla	7,176	LAW	LAW	LAW	LAW	LAW2	–
Eulipotyphla	5,537	LAW	LAW	LAW	LAW	LAW	LAW
Primates	2,390	LAW	LAW	LAW	(LAW)	LAW	LAW
Proboscidea	2,383	LAW	LAW	LAW2	LAW	–	–
Lagomorpha	2,326	LAW	LAW	LAW	LAW	LAW	Pow
Condylarthra	1,074	LAW	LAW	(LAW)	LAW2	–	
Chiroptera	853	LAW	LAW	LAW	LAW	LAW2	LAW
Cimolesta	597	LAW	LAW	LAW	LAW	–	–
Multituberculata	500	(LAW)	LAW	LAW	Pln	–	–
Creodonta	466	LAW	LAW	LAW	LAW	–	–
Didelphimorphia	312	Pow	LAW	LAW	LAW2	LAW	Pln2
Cingulata	219	LAW	Pln	LAW2	LAW2	Pln	–
Pilosa	175	Pln	LAW	(LAW)	LAW	–	–
Mesonychia	166	LAW	(LAW)	Pln	LAW	–	–
Notoungulata	148	Pln	–	LAW	–	–	–
Hyracoidea	147	LAW	Pln	Pln	Pln	–	–
Leptictida	126	–	Pln		Pln	–	–
Sparassodonta	74	LAW	–	LAW	–	–	–

“LAW” indicates that Type II pattern is best supported, “Pln” indicates that Type I pattern (plain) is best supported, and “Pow” indicates that Type III pattern (the “power law”) is best supported. “2” indicates that the best fit is on quadratic transformation (area rather than range width). Parentheses indicate that the best supported pattern is statistically weak, R^2^ < 0.9. The orders are sorted from the most abundant in the fossil record down to less abundant. Dashes indicate no or not enough data.

**Fig. 4.**
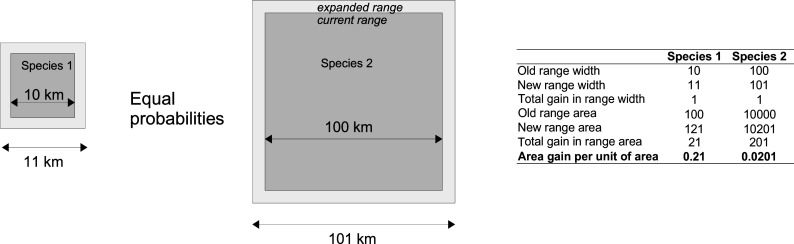
A schematic illustration of constant probability of expansion. We use squares for easier visual interpretability. The sizes are not to scale.

We can see from the table that for the most abundant mammalian orders Type II relationship (LAW) is best supported. These results suggest that similarly to Van Valen’s law of constant extinction, expansion in space follows memoryless decay, which relates to the range width. This implies that the probability of taxon’s range ever expanding by one step in space (e.g., by one km) does not depend on the range width the taxon currently occupies. For instance, a species that occupies a range of 10 km in width would have the same probability of expanding to 11 km as a species that has a range of 1,000 km would have of expanding to 1,001 km, as conceptually illustrated in Fig. 4. This expansion does not need to happen at the same time. The species can stop expanding at a given range size, which is from the analytic perspective analogous to extinction in survivorship curves, or continue expanding, which is from the analytical perspective analogous to species’ surviving depicted in survivorship curves. The main implication of this pattern is that under memoryless decay of expansion taxa are equally likely to expand no matter what range sizes they occupy.

Furthermore, while the difficulty of expanding remains constant over space, the returns per area are diminishing. In our example in Fig. 4, both species expanded their range width by 1 km. The areas that they gained (light gray in Fig. 4) were 21 km^2^ and 201 km^2^ correspondingly. Thus, species #1 gained more area in the absolute terms. However, if we consider how much each species gained in relation to the area that they already controlled, we see that species #1 gained 0.21 km^2^ per km^2^ it already controlled, and species #2 gained 0.0201 km^2^ per km^2^. Assuming comparable distributions of population densities, under linearly memoryless expansion process each individual contributes less and less to expansion as the species’ range grows.

## Hominins

Primates show a peculiar bimodal fit pattern in Fig. 3. This is mainly because the survivorship and expansion patterns of Hominini differ from the rest of the Primates (and the rest of the mammals analyzed). A more detailed analysis of scaling within Primates is presented in supplement. When Hominini are excluded from the analysis of Primates, the remaining Primates show statistically strong Type II pattern, which is in line with the majority of other mammalian orders.

In contrast, for Hominini the dominant pattern for range expansion appears to be Type III, whereas the dominant pattern for survivorship is Type I. Both are not memoryless. The scaling of survivorship implies aging for Hominini taxa, whereas the scaling of range expansion implies acceleration. In combination, these two scaling patterns could relate to specific competitive conditions outside the Red Queen’s theory. Pending further investigation, these patterns could offer insights into questions such as what processes have lead to a situation where only one species of humans remains today.

It is too early to conclude whether this pattern reflects true scaling of hominins or it is more due to overenthusiastic taxonomic splitting of hominin species in the research community. The exceptional scaling of hominins manifests itself stronger when using the inclusive taxonomic treatment of Hominini, whereas using a conservative taxonomic treatment (30) brings the scaling patterns closer to the memoryless scaling, common for other mammals. Nonetheless, even with the conservative taxonomic treatment we cannot reject that the real scaling of Hominini is exceptional, as our supplementary analysis demonstrates. The result is in line with a recent study (31), which showed unusual and unexpected patterns of diversity dependence in *Homo* speciation and extinction.

While the datasets for Hominini are small and uncertain and the model fits for this group lack robustness; if these scaling patterns for hominins reflect the reality, this would imply different laws of competition for hominins rather than the classical Red Queen’s competition (1, 7). Pending further investigation, this would have interesting implications for early human evolution research and beyond.

## Interpretations

Apart from the interesting discourse for Hominins, our results suggest that the most common macroevolutionary species expansion in space is memoryless similarly to Van Valen’s law that describes species survival in time. A question arises how the new patterns of constant expansion relate to the original law of constant extinction. Is the same process driving both? And is one predictable from the other?

Species durations in the fossil record are only very mildly correlated with the maximum range sizes that species occupy. Our supplementary online repository presents the statistics for this relationship (https://github.com/zliobaite/expansion). Indeed, we know that there are long-lived widespread taxa in the fossil record, but there are also widespread taxa that are short-lived as well as there are long-lived taxa that never become widespread (32). There are taxa that expand rapidly, and there are taxa that expand slowly (15). Our earlier mechanistic modeling (20) suggests that evolutionary patterns consistent with the Red Queen’s hypothesis can be generated assuming that the potential to expand is internal to a species but the stop of expansion comes externally from competition. This can happen in a rapidly changing environment, yet changes in the environment are not mandatory for this process to work, which is in line with Van Valen’s reasoning (1).

While we see that the aggregated patterns of survival in time and expansion in space are consistent with the Red Queen’s hypothesis as a stochastic macroevolutionary process, this does not mean ecology and environment do not matter for survival or expansion. In the Red Queen’s realm extinction occurs randomly with respect to age but not randomly with respect to ecology (1). Similarly to the law of constant extinction, we expect the patterns of constant expansion to hold under comparable ecological circumstances, which can be approximated as adaptive zones (20, 21). Even each species faces individual pressures and circumstances, when considered together, we see consistent large-scale patterns emerge.

Following the metabolic scaling, the ranges of individuals, just like the expected longevity of individuals, scale positively with body mass (33). At the species level, the life expectancy or maximum range size of a species is effectively independent of the body mass of individuals. Our supplementary online repository reports the statistics on the mammalian fossil record. These patterns are consistent with the Red Queen’s theory (1, 20); in which species is the unit of competition that controls a stochastically constant amount of energy at any time. If species having larger individuals, all else being equal, occupied larger range sizes, this would imply that such species can control more energy. If true, this premise would suggest that larger species have an overarching competitive advantage. Instead, the Red Queen’s theory accommodates (20) how the species of small body sizes can compete with species of large body sizes, young species can compete with old species, and species occupying small ranges can compete with species occupying large ranges.

Perhaps the most intriguing result in relation to range expansion is that the fit of expansion curves is better over the linear dimension of the maximum range size rather than its area. This implies that species expand in steps proportional to their range width. Since the perimeter of a range is proportional to the range width, we interpret this pattern as evidence that macroevolutionary expansion happens primarily via the perimeter of a species range. This is consistent with the leading-edge expansion theory (34) from ecology. This pattern is also consistent with the results from mathematical modeling of animal dispersal and biodiversity gains (35, 36).

The original Red Queen’s hypothesis has not been explicit about the mechanisms of expansion in species’ range or its abundance; our analysis offers an opportunity to link those mechanisms to the Red Queen’s theory. Our observed patterns of constant expansion in proportion to the range width imply that relative gains of area are not constant. While each species’ expansion step gives access to a larger area and thus brings more energy to be controlled in absolute terms, relative gains in expansive energy are diminishing.

The energy controlled by a species is a quadratic characteristic (it relates to the range area), but the expansive energy is linear (it relates to the range perimeter). This mismatch of scales in the energy controlled and the expansive energy can be interpreted as the inertia that prevents species from expanding or going extinct instantaneously (7). Indeed, species that have larger ranges have been shown to have lower extinction probabilities (16, 37, 38). Thus, even if species’ expansion in space and survivorship in time follow memoryless decays and more time does not guarantee access to more space, more space may give more time.

Overall, we have learned from the analysis that macroevolutionary expansion in space is memoryless in relation to the space already occupied by taxa. Memoryless decay does not mean that all taxonomic lineages have equal probabilities of expanding or going extinct. Rather, this means that in comparable ecological contexts, having existed long enough does not help to prevent extinction, and similarly, already controlling a large range does not help to accelerate expansion. Since the expansion steps are linear, expansion must primarily happen through the perimeter rather than through all the species range area. And when the steps are linear, the gains in area diminish with the range size. Thus, even if a taxon can potentially live for a very long time, it cannot expand indefinitely, even in the memoryless Red Queen’s realm.

## Methods

Our main computational task is to statistically distinguish between three types of survivorship curves. Then, for each mammalian order in the fossil record, we can ask whether survivorship of species (or genera) follow Type I, Type II, or Type III patterns, as well as whether maximum ranges in space follow Type I, Type II, or Type III pattern. Table 2 summarizes possible outcomes and their corresponding interpretations.

**Table 2.** Possible outcomes of the analysis and their interpretations

	If Type I relationship fits the data best	If Type II relationship fits the data best	If Type III relationship fits the data best
Taxon survivorship	Taxa age – extinction risk increases when taxa get older.	Van Valen’s law of constant extinction holds – the extinction risk does not depend on taxon’s age.	Rich get richer – those that already survived long are more likely to survive even further.
Taxon maximum ranges	Expansion saturates – those that have larger ranges are less likely to expand further.	Constant Expansion holds – the probability to expand in range does not depend on how large of a range the taxon already occupies.	Rich get richer – those that already have large ranges are more likely to expand further.

For each mammalian order in the global fossil record throughout the Cenozoic, we test whether Type I, type II, or type III has the strongest statistical support. We test this for survivorship, as in the original formulation of the law of constant extinction, as well as for maximum ranges of taxa. We test the scaling of expansion in relation to the linear representation of the taxon’s range (range width) as well as in relation to the quadratic representation of the taxon’s range (range area) in the fossil record.

Following the same statistical methodology we separately test the scaling of the present-day ranges of mammalian species and genera. We test the scaling with the present-day as well as paleocoordinates of the fossil localities. We test the scaling with four alternative range shapes. To complement the analysis, we run a probabilistic simulation of the fossil record to investigate how survivorship curves behave under potential preservation biases.

### Analytical Tests.

**Fig. 5.**
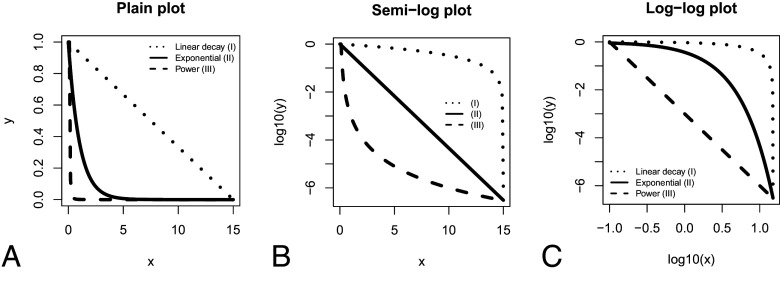
Three types of decay models plotted in (*A*) plain, (*B*) semilog, and (*C*) log–log plots. The exact models in this example are linear y = 1− 0.0666666x; exponential y = e^−x^; power y = 0.001×^−3^.

The key to our testing is that each of the three relationships (Type I, Type II, or Type III) plot linearly at different standard transformation of the coordinate system. Fig. 5 shows transformations of three base models in three coordinate systems: plain, semilog, and log–log.

A linear decay model takes the form **y** = b – a**x** and gives a straight line in the linear coordinate system. An exponential decay model takes the form **y** = ce^−d^**^x^** and is a straight line in the semilog plot. A power law decay takes the form **y** = g**x**^−h^ and gives a straight line in the log–log plot. Here, **x** is the input variable (taxon’s duration or range size), **y** is the dependent variable (the proportion of taxa from the initial sample surviving or expanding at each step in time or space), e is a mathematical constant (Euler’s number), and a, b, c, d, g, h are parameters taking different values to fit the relationships to the data.

Our test on the fossil data is thus very simple. We transform the survivorship and separately the maximum range data into these three coordinate systems: plain plot (no transformation), semilog transformation, and log–log transformation and test which transformation gives the best linear fit.

Our tests address only three basic types of relationships, certainly, the reality can follow combinations of those, which can be interpreted as, for instance, Type II-and-a-half. We do not consider such partial types within the scope of this study. Our goal is to assign the relationship to the closest one of the three basic types.

We use ordinary least squares regression for this purpose and use the coefficients of determination (R^2^) to select the best fit. Due to non-independence of observations R^2^s turn out quite high in their absolute values; however, nonindependence affects data in the same way in all three transformations, thus relative comparisons of the statistics are plausible. It does not matter for the test which logarithm base to use, thus we use log_10_ for best human readability of the plots.

**Fig. 6.**
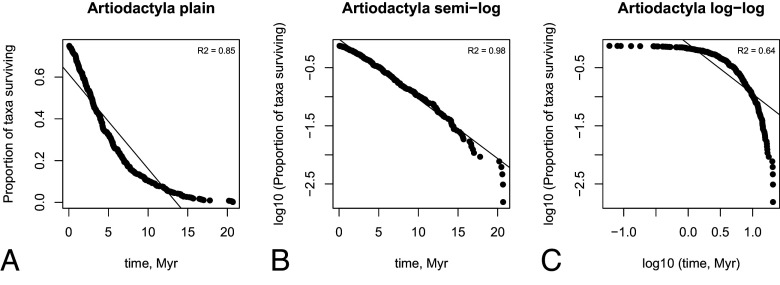
Testing which type of decay fossil genera follow in the order Artiodactyla. (*A*) plain plot for range width, (*B*) plain plot for range area, (*C*) semilog plot for range width, (*D*) semilog plot for range area, (*E*) log-log plot. The *Top Right* corner shows the coefficient of determination (R^2^).

Fig. 6 illustrates the test for survivorship of genera in the order Artiodactyla in the fossil record. We see that semilog plot visually as well as using R^2^ statistics shows the best fit. Thus, we conclude that the law of constant extinction holds. We do this test for each order at the species level as well as separately at the genus level and report the best fits in Table 1 in the main text. Individual plots for all the orders are given in the online repository (https://github.com/zliobaite/expansion).

Similarly to survivorship, we test which type of relationships maximum ranges of taxa follow. Instead of time we now have space on the horizontal axis. For each value on the space axis, we plot the proportion of taxa that have a larger range size than that threshold. This treatment is similar to the analysis of survivorship, but here the plots show the proportion of taxa that expand beyond a particular range size. At each threshold of the range size on the horizontal axis taxa fall into two groups—those taxa that have stopped expanding (the maximum observed range size is smaller or equal to the threshold) and those that will continue expanding (the maximum observed range size is larger than the threshold). We do not need to follow taxa over time for this analysis of expansion in space. Sufficient is to know that if the maximum observed range of a taxon is larger than the current threshold, the taxon must have expanded, no matter how long it takes to expand to the next range size.

This analysis of expansion differs from the analysis of survival in a nuanced way. The duration in time is a linear characteristic, whereas range area in space is a quadratic characteristic. To account for possible different types of processes, we investigate two variants of range expansion—linear and quadratic. We refer to the linear variant as the range width to be consistent with our fossil range data primarily handled as rectangular ranges. We use the square root of the range area as the measure of the range width. More generally one can think of this linear characteristic as the diameter of the range, where the range can be approximated as a rectangular, a circle, an ellipse, or a convex hull.

To analyze expansion in space, we assess model fits over five transformations. We project the data into plain, semilog, and log–log coordinate systems, and we do this for the range width and the range area separately. In the log–log transformation the change from range width to range area is already linear, thus we only keep one variant, hence we have five candidate transformations, rather than six. Similarly, as for survivorship, we assess which of the transformations gives the best R^2^.

**Fig. 7.**
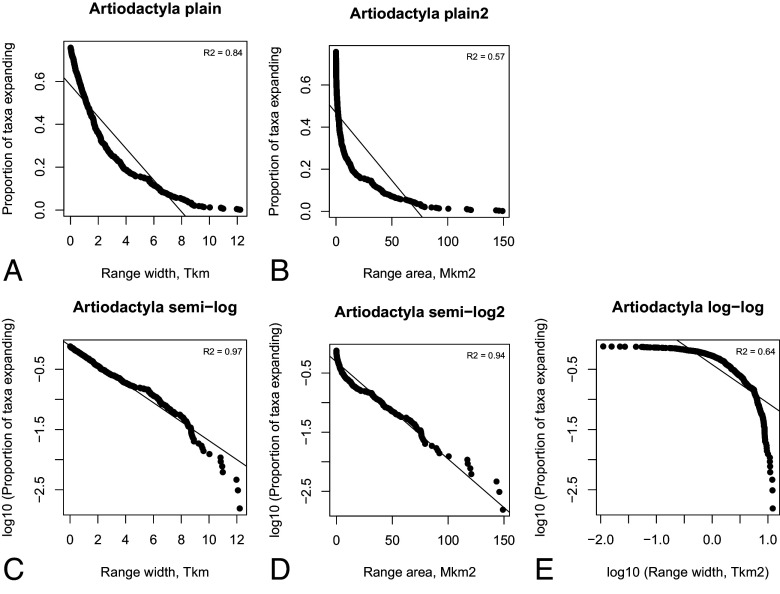
Testing which decay ranges of genera in the order Artiodactyla follow in the fossil record. The *Top Right* corner shows the coefficient of determination (R^2^).

Fig. 7 illustrates the procedure for the order Artiodactyla in the fossil record. We see that the semilog plot over the range width on the horizontal axis gives the best fit. The semilog plot over the range area gives a good fit as well, but we see visually that at low values on the horizontal axis the curve is bent. A good fit to that part of the data is more critical, since there are many more data points at small ranges. From the observation that semilog plot with range width gives the best fit, we conclude that the probability of expansion by one linear step in range is memoryless with respect to how large of a range a taxon already has. We report additional tests with paleocoordinates as well as different shapes of ranges in supplement text as well as in the online repository (https://github.com/zliobaite/expansion).

The results for all the major mammalian orders in the fossil record and at the present-day are presented in Table 1 in the main text. We report this for every order that gives at least 10 data points for the analysis. We analyze the scaling separately at the genus level and at the species level. With the fossil data, we analyze the scaling of durations and ranges, with the present-day data we only analyze ranges. Plots illustrating fits for each order separately are available in the online repository (https://github.com/zliobaite/expansion).

### Mathematical Interpretations of Three Survivorship Curves.

Type II survivorship curve, which is linear in a semilog plot (Fig. 5), is mathematically described as exponential decay. Exponential decay is typically used to model, physical, chemical, or social processes, and, most famously, the decay of radioactive isotopes. The amount of substance decreases at a constant rate in proportion to the amount remaining. This can be expressed via a differential equation as dN/dt = −λt. The solution to this equation is N_t_ = N_0_e^−λt^, where N_t_ is the quantity at time t, and N_0_ is the starting quantity. Thus, when the number of taxa surviving over time follows this relationship, the probability of survival to the next time step can be expressed as

P_t_^survival^ = N_t+1_/N_t_ = N_0_e^−λ(t+1)^/N_0_e^−λt^ = e^−λ^. We see that this probability does not depend on time (t). As the probability of survival does not depend on t, the probability of extinction (1 − P_t_^survival^) also does not depend on t.

Type I survivorship curve is linear or closest to linear in the plain plot (Fig. 5). The number of taxa alive in this setting can be modeled as a linear regression N_t_ = N_0_ − ct, c is a parameter. In this case, the amount vanishing at each step is constant not in relation to the amount that remains, but in relation to the initial amount. The probability of survival here can be expressed as

P_t_^survival^ = N_t+1_/N_t_ = (N_0_ − ct − c)/(N_0_ − ct) = 1 − c/(N_0_ − ct). We see that this time the probability of extinction depends on time (t). If t goes up, the second term will go up, thus the probability of survival will go down. This implies aging—the larger the age, the lower the probability of survival is.

Type III survivorship curve is linear or closest to linear in the log–log plot (Fig. 5). This corresponds to power law, popularly known by many terms including rich get richer or “80-20” rule. Power law is particularly used to model phenomena that relate to behavior, for example, the distribution of wealth or the distribution of clicks or likes on the web. The power law is also commonly used to model scaling of living organisms. A model form for power law survivorship is N_t_ = bt^−k^. In this case, the probability of survival depends on time (t), since P_t_^survival^ = N_t+1_/N_t_ = ((t + 1)/t)^−k^. We can see that if t goes up, the probability of survival will go up. This implies that the chances for survival improve with age.

The reasoning regarding the distribution of maximum ranges in space is similar to the reasoning regarding the distribution of durations in time. The probability of expansion in Type I and Type III relationships depends on the range size that a taxon currently occupies, while in Type II relationship, the probability of expansion is constant in relation to the range size that is already occupied.

### Fossil Data and Preprocessing.

Our analysis covers the global fossil record of mammals throughout the Cenozoic, publicly available through the NOW database of fossil mammals (29). The reported results are over a full public version of the NOW database, downloaded on the 18th of August 2023.

Our main analysis of the fossil data is at the species level since species is the primary unit of evolutionary expansion. For comparability to the present-day analysis, we carry out the analysis at the genus level, which is known to be more robust for macroevolutionary and paleoecological analyses of the mammalian record than the species level (28). We report results both ways.

We use occurrences of taxa that are identified to the species or genus level in the database correspondingly. Following the common practice of macroevolutionary analysis of the NOW data we discard “indet” and unnamed but keep those identified with uncertainties such as “cf” or “aff”. We include occurrences from the localities where the age range does not exceed 3 My, we discard localities having broader age ranges, which can be either due to time averaging or due to uncertainties in dating. This threshold is chosen roughly as the minimum threshold that allows not to discard localities dated by the mammalian land zones, especially in the Miocene Europe, which comprise a notable part of the mammalian fossil record. While generally the age resolution in the database gets more precise closer to the present-day, for comparability we keep the selected age threshold constant through the Cenozoic.

For each taxon (genus or species, depending on the variant of the analysis) we need to know how long it existed in time and what was its maximum range in space. To retain as much information as possible we work with real-valued durations in this analysis rather than assigning species or localities to time bins. We calculate the duration in time as the maximum difference between mid-points of age ranges of the localities at which the taxon occurs.

We approximate the maximum range of a taxon as a rectangular bounding box resting on the minimum and the maximum latitude and longitude of the localities at which the taxon occurs. A schematic illustration of this process is shown in supplement (*SI Appendix*, Fig. S2). The range defined this way does not have to be occupied at the same time, although in many cases it is. This treatment allows us not to force localities into time bins, since we aim at analyzing patterns in space separately from analyzing patterns in time. We acknowledge that ranges in reality are rarely rectangular and they may be discontinuous in space, for instance, they may include water. A rectangular shape represents the upper bound of the range and works well for robustness and consistency of treatment. We include a complementary analysis with ellipses, convex hulls, and geospheres in supplement. The resulting patterns are similar and the main conclusions hold under all the variants.

In the main analysis for each taxon, we compute the area of the rectangular range. The range width is then computed as the square root of the area. We assume that one degree in latitude or longitude is equal to 111 km. Degrees of latitude on Earth are 60 nautical miles apart. One degree of longitude at the equator is equal to 60 geographical miles, which is very similar in length to the nautical mile, it equals to 1,855 m. This gives 111 km for each degree of longitude at the equator. Longitude gets smaller toward the poles until the distance between degrees of longitude becomes 0 km at the poles. Most of the mammalian fossil record is closer to the equator than the poles. For simplicity, in the analysis, we approximately treat the pixels of the longitude-latitude as squares. Supplement includes a variant of the analysis with more precise conversion of the longitudinal degrees to kilometers, the principal patterns are very similar and the main conclusions hold. We do not explicitly handle the collision between −180 and 180° of longitude since there is no terrestrial mammalian fossil record around that area.

In the main analysis, we use the present-day coordinates of localities for spatial analysis. From the macroevolutionary perspective we are interested in the species potential to expand. Suppose that a species does not expand at all, but their continental plate moves and thus the coordinates of their range change. If the coordinates move apart, this could increase a perceived maximum range even no competitive expansion has taken place. Tectonic movements can introduce noise in this analysis if a species occupies a range over several tectonic plates that move in different directions, yet this primarily affects very large ranges. Our experiments with eight tectonic models available through rgplates (39) revealed that different projections of present-day coordinates into paleocoordinates largely diverge. Given that the continents have not moved too much through most of the Cenozoic and that we are interested in the extent of competitive expansion rather than the absolute position of the range, we use the present-day coordinates for the main analysis. We report two variants of analysis with paleocoordinates in supplement. The principal patterns are very similar to the main analysis and the main conclusions hold.

We analyze patterns of macroevolutionary decay by orders, one order at a time. This is to approximate adaptive zones of mammals. While the original concept of an adaptive zone was not taxonomic (40), for the operational purposes, higher taxonomic units were used as an approximation (1) of a similar resource space and ways of life (21). We thus use orders to approximate adaptive zones.

The Table 3 shows a small selection of genera from order Artiodactyla to illustrate the protocol of assembling datasets for fitting models for each order. In the real analysis, we include all the genera available from each order, thus the actual series used in the analysis are much longer. First, we list the durations of each species (or genera) that belong to that order as exemplified in Table 3. We sort unique durations from the shortest to the longest (Table 3*a*). We then calculate how many genera survive longer than each time threshold (Table 3*b*). We discard the first and the last rows for robustness and use the remaining rows as input to the model fitting. The first zero is not really a zero duration but rather an artifact of having singletons in the fossil record (a taxon that occurs only once). The last zero is where history ends, since species are discrete units, and we cannot have fractional species. This would cause problems when taking logarithms. In the main text, we report the results for the orders that give at least 10 distinct time points after discarding the first and the last zero.

**Table 3.** An example of dataset assemblage

(a)		(b)	
Genus	Duration, My	Time, My	Proportion surviving
*Ammotragus*	0	0	0.7
*Sinoryx*	0	0.18	0.5
*Toromeryx*	0	1.45	0.4
*Proamphibos*	0.18	2.62	0.3
*Probison*	0.18	3.03	0.1
*Selenoportax*	1.45	3.12	0
*Urmiatherium*	2.62		
*Bison*	3.03		
*Megaloceros*	3.03		
*Pelorovis*	3.12		
(c)		(d)	
*Genus*	Maximum range width, thousand km	Range width, thousand km	Proportion expanding
*Ammotragus*	0	0	0.8
*Sinoryx*	0	0.07	0.6
*Proamphibos*	0.07	0.35	0.5
*Probison*	0.07	2.17	0.4
*Toromeryx*	0.35	2.21	0.3
*Selenoportax*	2.17	6.13	0.2
*Urmiatherium*	2.21	6.56	0.1
*Pelorovis*	6.56	12.21	0
*Bison*	12.21		

This is based on a small selection of genera from order Artiodactyla

The Table 3 *c* and *d* illustrate data assemblage procedure for analysis or ranges. The first zero is not a zero-sized range in reality but an artifact of having singletons in the fossil record (a taxon that occurs only once in the record). Those species exist in the record and thus are needed to estimate the fraction of surviving or expanding species out of the total number of relevant species in the fossil record. The species with zero ranges are excluded from the reported survivorship curve fitting.

The analysis of ranges is independent from the analysis of survivorship. Indeed, we can see that the order of genera from the smallest to the largest duration is not the same as the order from the smallest to the largest range.

### Potential Preservation Biases.

We reason about evolutionary processes of the living from the fossil record which is sparse and incomplete in terms of represented species (41). We thus run a simulation to check what happens to the survivorship and expansion patterns if species survivorship and range sizes follow Type II relationships but only very few individuals out of those that are alive have a chance to fossilize. In these circumstances the species that have been more widespread and longer-lived are more likely to make it to the fossil record. The goal of this simulation is to investigate whether we would still see the underlying laws in the fossil record formed in this way.

Our randomized simulation is as follows. We have a world where individuals of each species are of the same size and thus have the same life expectancy and the same population density as per metabolic scaling laws (33). In this simulation, we assume that the expected lifespan of an individual is 14 y and the population density is 10 individuals per km^2^. This roughly corresponds to adaptive zones of large herbivores with the average adult body size of 75 kg. We can think of a goat as an example mammal within this category.

We use a constant speciation rate, a new species arises every 2000 y. Durations of species are drawn from the exponential distribution with the mean species duration of 3.33 My. Peak ranges of species are also drawn from the exponential distribution with the mean of 694 km^2^. We assume that the peak range is reached in the middle of species duration in time and the rise and decline to the peak is linear.

Within each range, we assume a uniform population density and thus we can calculate absolute abundances knowing the range sizes. Then, for each individual within the abundance we assume a constant lifespan as per life expectancy defined above. When an individual dies in the model, it is immediately replaced by a new individual. Following this process in the model we can count of how many individuals die per area per species per year. We then assume that when each individual dies it can fossilize and make it to the fossil record with a fixed probability, we find that the probability of 10^−11^ to 10^−13^ gives us simulations with realistically looking fossil assemblages.

We assume that those individuals that fossilize and are exposed and found are perfectly identifiable to the species level and all of them make it to the fossil records. We can infer species durations from the first and the last occurrence of individuals belonging to that species in the model.

**Fig. 8.**
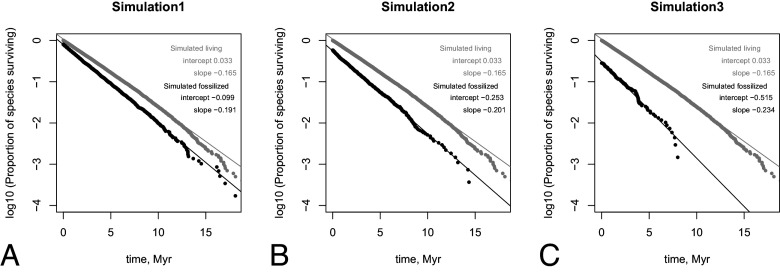
Simulation results with three different fossilization rates. The gray dots and lines give survivorship curves if we observed all the individuals that have lived; those dots and lines are identical in all three panels. The black dots and lines give survivorship curves with the following fossilization rates: Simulation1 assumes that 1 in 10^11^ living individuals make it to the fossil record, Simulation1—10^12^ and Simulation3—10^13^.

We run a simulation for a total of 12 million simulated years. We then compare the survivorship curves to be inferred from presumably living individuals in the simulation with curves inferred from a presumed fossil record. Fig. 8 gives simulation results with three different fossilization rates.

We can see from the plots that even if longer-lived and wider-spread species in the simulation are more likely to make it to the fossil record, we clearly recover linear survivorship curves in semilog plots. We thus conclude that even if preservation is biased toward more widespread and longer-lived species, fundamental shapes of survivorship laws can be recovered.

There is an interesting analytical potential in the interpretation of the intercept. The intercepts from fits to the real fossil record can potentially be used to estimate the preservation rate. The smaller the intercept, the lower is the preservation rate. Analytical treatment of this will be addressed separately elsewhere.

### Present-Day Data and Preprocessing.

Our present-day species occurrence data draw on the ranges from The IUCN Red List of Threatened Species (42), processed in Phylacine (43) atlas and projected onto a hexagonal grid (44) with grid cells of about 50 km in diameter. We consider all the mammalian orders. We exclude aquatic mammals (order Sirenia and clade Pinnipedia). Trait data for present-day come from Phylacine (43).

We use two ways of estimating species range sizes. The main way, used for the analysis reported in the main text, is the same as for the fossil data. We put a bounding rectangular box that rests on the minimum and the maximum latitude and longitude of the hexagons where the taxon occurs. We use the coordinates of the center of the hexagon. We do not explicitly handle the collision between −180 and 180° of longitude. We estimate the range width taking the square root of the range area. The second way to estimate the range is to count the number of hexagons where the species is present and multiply it by the area of a hexagon (1,643 km^2^). When analyzed this way the main results are similar with a bit more variation of prevailing patterns at the genus level. The results of this analysis are available in the online repository.

One major difference between the fossil data and the present-day data is that in the fossil record, we have maximum ranges over the existence of a species, whereas at present-day, we only have current ranges, which for some species may be the maximum ranges they will ever achieve, but for other species, they are interim ranges. For analysis of survivorship, this situation would be referred to as censored data. There is a major difference, however, which makes analysis of ranges easier. Durations always increase over the lifetime, whereas ranges first increase and then decrease (7), peaking somewhere in the middle of a species lifetime. Assuming roughly unimodal patterns of rise and decline through the species lifetime (6), we can think that in the expectation each species occupies roughly half of the range that they would ever achieve. If that is the case, the large-scale range distribution patterns should still be recognizable, even if they are noisy.

For the present-day, we are thus estimating the lower bound of the probability of expansion. We can think of two extreme scenarios for interpreting the present-day data. Scenario 1: the ecosystems are at the equilibrium and thus the observed ranges mostly represent the peak ranges. Scenario 2: the ecosystems are dynamically as far from the equilibrium as possible thus assuming continuous expansion and decline each taxon is about mid-way through to or from its peak range. In Scenario 1 we can consider the observed probabilities as true probabilities of expansion. In scenario 2, even if the absolute probabilities are not precise, we can still reason about the shape of the relationship (Type I, Type II, or Type III) even the absolute values of the probabilities reflect the lower bound.

The following additional simulation supports the argument. We simulate species duration and range data in the following way. At each time step one species originates. After originating species expands either one unit of range width per time step (Case A) or one unit of range area per time step (Case B). Case A corresponds to our main conclusion that expansion happens via the frontier of the range. Case B corresponds to the scenario that expansion happens from within the whole range area. The main goal of this simulation is to check whether our statistical test can distinguish between Case A and Case B in case we only have observational data from one time point, such as the present-day mammalian data. The test results for this simulation are given in Table 4.

**Table 4.** Coefficients of determination (R2) of Type I, Type II, and Type III model fits on simulated species ranges when decline starts at a random point in the species duration as per Red Queen’s hypothesis: a) statistical tests for alternative model fits when peak ranges are considered (similarly to the fossil data); b) statistical tests when ranges are observed at one point in time and they are not necessarily at peak (similarly to the present-day data)

a) Peak ranges		R2 Type I	R2 Type II	R2 Type III
Case A expansion by the range frontier	Model fit: probability of expansion vs. range width	0.724	0.994	0.782
	Model fit: probability of expansion vs. range area	0.443	0.926	0.782
Case B expansion by the range area	Model fit: probability of expansion vs. range width	0.879	0.937	0.782
	Model fit: probability of expansion vs. range width	0.724	0.994	0.782
b) Snapshot ranges		R2 Type I	R2 Type II	R2 Type III
Case A	Model fit: probability of expansion vs. range width	0.877	0.978	0.668
	Model fit: probability of expansion vs. range area	0.585	0.907	0.668
Case B	Model fit: probability of expansion vs. range width	0.882	0.971	0.668
	Model fit: probability of expansion vs. range width	0.877	0.978	0.668

In both panels a and b, the first and the fourth lines are the same because the results are drawn from the same simulation (with the same random seed) while the interpretation of the units is different: either km or km^2^.

In case A, we see that Type II relationship, which corresponds to the proposed law, shows the best fit. This is because we simulated the data following the process corresponding to the law of constant expansion. Yet, constant expansion can be via the frontier or via the whole range area. If expansion happens via the frontier (Case A), we expect the probability of expansion vs. range width shows a better fit than the probability of expansion vs. range area. The results in Table 4*a* support that. If expansion happens via the range area (Case B), we expect the opposite and the results Table 4*a* also support that.

The results in Table 4*b* are similar conceptually but with weaker fits. This supports the expectation that snapshot data can capture patterns as proxies to the peak data. The reported results are over one point in time. We tested with different points in time, the results are consistent.

The code and plots for this simulation can be found in folder 6_present_day_simulations in our online repository.

## Supplementary Material

Appendix 01 (PDF)

## Data Availability

All the data used, analysis code, as well as supplementary figures are available in an online repository: https://github.com/zliobaite/expansion (45).
