# Supplementary material for: Laws of macroevolutionary expansion
Source: Proc Natl Acad Sci U S A. 2024 Aug 6;121(33):e2314694121. doi: 10.1073/pnas.2314694121 (PMC11331108; doi:10.1073/pnas.2314694121)
Supplement: Supplementary file 1 — Appendix 01 (PDF) [file pnas.2314694121.sapp.pdf]

Supplement to: Laws of Macroevolutionary Expansion

Indrė Žliobaitė

University of Helsinki, Finland
Email: indre.zliobaite@helsinki.fi

2024 05 26

Scaling of durations and range expansion for Primates

Primates show a peculiar pattern of scaling for range expansion in the fossil data. The pattern for range looks like a broken stick (Figure 3 a) in the main text). The slope gets shallower at larger ranges. The largest ranges in Primates are occupied by *Homo sapiens, H. erectus and H. heidelbergensis*, followed by *Theropithecus oswaldi* (occurrences ranging from South Africa to Spain). *H. sapiens* is not plotted since the largest and the smallest range widths are removed before curve fitting as per protocol outlined in Table 3. While the data inevitably contains uncertain occurrences that might exaggerate ranges in general, in all these cases occurrences implying large ranges appear to be quite certain. In this supplementary analysis we look closer into scaling of durations and range expansion for Primates.

We split Primates into the following subgroups:
1) tribe Hominini;
2) family Hominidae excluding tribe Hominini;
3) the remaining Primates excluding family Hominidae.
In this supplementary analysis we consider two variants of tribe Hominini – an inclusive species list and a conservative species list. We follow the taxonomic treatment of Martin et al. (1) to form those lists. The taxonomic variants that we use are given in Table S1.

Our primary results reported in the main text are following the taxonomic treatment of the NOW database, which very closely corresponds to the inclusive list of Martin et al., as reported in Table S1. NOW additionally has *Homo rhodesiensis,* which we synonymize under *Homo heidelbergensis* to match with the taxonomy of Martin et al. in this supplementary analysis. NOW data mises occurrences of six newest species of Homo, listed in the inclusive list of Martin et al.

We fit scaling models to the subgroups of Primates following the protocol outlined in the Methods section. We must lower the minimum points for curve fitting from 12 to 9 in order to be able to include the conservative variants of hominin taxonomy in the analysis.

Table S2 shows the statistics of alternative model fits for species survivorship and expansion of their ranges. Figure S1 shows the corresponding plots. We can see that Type II models show the best fits for all the groups except for Hominini. Type II model corresponds to memoryless decay consistent with the Law of Constant Extinction (2) and constant expansion analyzed in this study. Type II scaling dominates in most other mammalian orders analyzed in this study.

It turns out, thus, that a relatively poor model fit for Primates is mainly because the scaling patterns of Hominini differ from the rest of the Primates. This exceptional scaling manifests itself more strongly when using the inclusive taxonomic list, while using the conservative list brings the scaling close to Type II scaling, common for other mammals. Yet, even with the conservative taxonomic treatment we cannot reject a possibility of exceptional scaling, as the following analysis demonstrates.

Table S1. Variants of taxonomic treatment of Hominini used in the analysis.

Hominini species in NOW	Martin et al (2024) long list	Martin et al (2024) conservative
Ardipithecus kadabba	*Ardipithecus kadabba*	*Ardipithecus ramidus*
Ardipithecus ramidus	*Ardipithecus ramidus*	*Ardipithecus ramidus*
Australopithecus afarensis	*Australopithecus afarensis*	*Australopithecus afarensis*
Australopithecus africanus	*Australopithecus africanus*	*Australopithecus africanus*
Australopithecus anamensis	*Australopithecus anamensis*	*Australopithecus afarensis*
Australopithecus bahrelghazali	*Australopithecus bahrelghazali*	-
Australopithecus garhi	*Australopithecus garhi*	*Australopithecus afarensis*
Australopithecus sediba	*Australopithecus sediba*	*Australopithecus africanus*
Homo antecessor	*Homo antecessor*	-
Homo cepranensis	*Homo cepranensis*	-
Homo erectus	*Homo erectus*	*Homo erectus*
Homo floresiensis	*Homo floresiensis*	*Homo floresiensis*
Homo habilis	*Homo habilis*	*Homo habilis*
Homo heidelbergensis	*Homo heidelbergensis*	*Homo heidelbergensis*
Homo luzonensis	*Homo luzonensis*	-
Homo neanderthalensis	*Homo neanderthalensis*	*Homo neanderthalensis*
Homo rhodesiensis	-	-
Homo rudolfensis	*Homo rudolfensis*	*Homo rudolfensis*
Homo sapiens	*Homo sapiens*	*Homo sapiens*
Kenyanthropus platyops	*Kenyanthropus platyops*	*Kenyanthropus platyops*
Orrorin tugenensis	*Orrorin tugenensis*	-
Paranthropus aethiopicus	*Paranthropus aethiopicus*	*Paranthropus boisei*
Paranthropus boisei	*Paranthropus boisei*	*Paranthropus boisei*
Paranthropus robustus	*Paranthropus robustus*	*Paranthropus robustus*
Sahelanthropus tchadensis	*Sahelanthropus tchadensis*	-
Not in NOW	*Homo ergaster*	-
Not in NOW	*Homo gautengensis*	-
Not in NOW	*Homo naledi*	*Homo naledi*
Not in NOW	*Australopithecus deyiremeda*	-
Not in NOW	*Homo longi*	-
Not in NOW	*Homo bodoensis*	-

Table S2. Alternative model fits to the global fossil record throughout the Cenozoic (a) species survivorship and (b) expansion of their range widths. Bold face indicates the maximum R^2 per group. R^2 denotes the coefficients of determination for linear model fits in the transformed space as outlined in Figure 7. The higher R^2, the better.

	N species for modelling	R^2 Type I (plain) Aging	R^2 Type II (semi-log) Red Queen	R^2 Type III (log-log) "Rich gets richer"
**(a) Species survivorship**				
Primates all	526	0.700	**0.972**	0.623
Primates not Hominidae	462	0.710	**0.970**	0.639
Hominidae not Hominini	40	0.856	**0.963**	0.853
Hominini inclusive	24	**0.967**	0.904	0.474
Hominini conservative	13	**0.916**	0.914	0.887
**(b) Species range expansion**				
Primates all	526	0.461	**0.850**	0.782
Primates not Hominidae	462	0.611	**0.931**	0.738
Hominidae not Hominini	40	0.687	**0.930**	0.896
Hominini inclusive	24	0.536	0.827	**0.877**
Hominini conservative	13	0.697	**0.896**	0.874

Since the smallest and the largest valued points are removed when fitting the models, results on species ranges do not include H. sapiens. Results for range areas are not included since in all cases they give worse fits than range widths.

(a) species survivorship

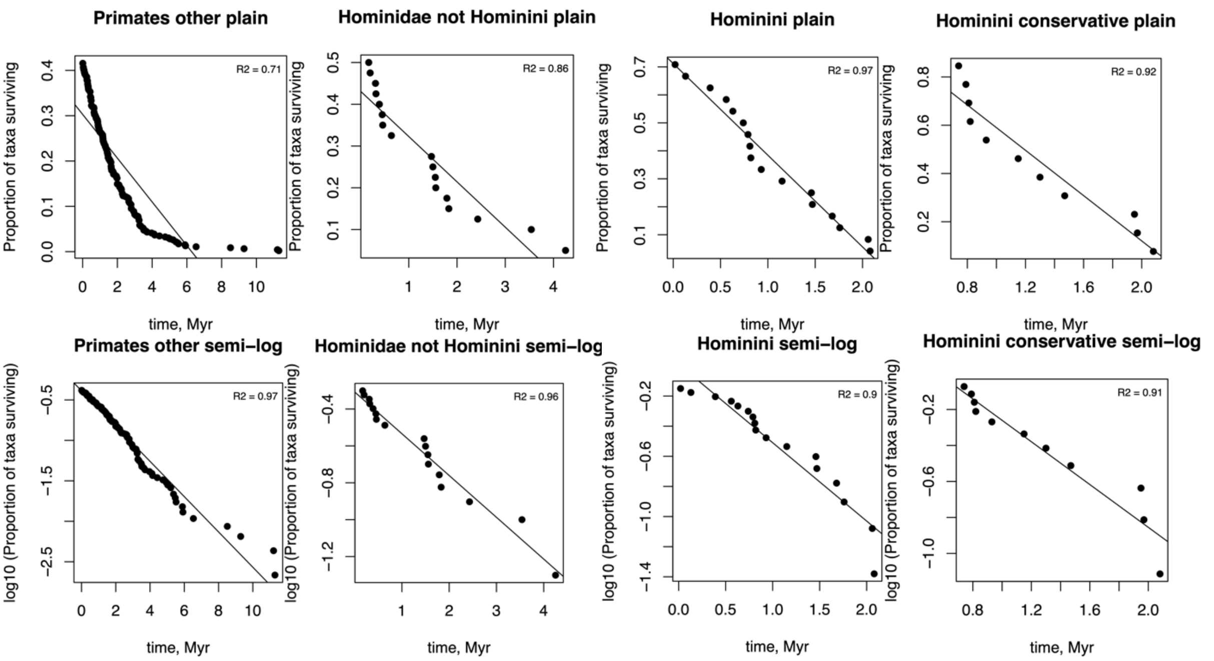

(b) range expansion

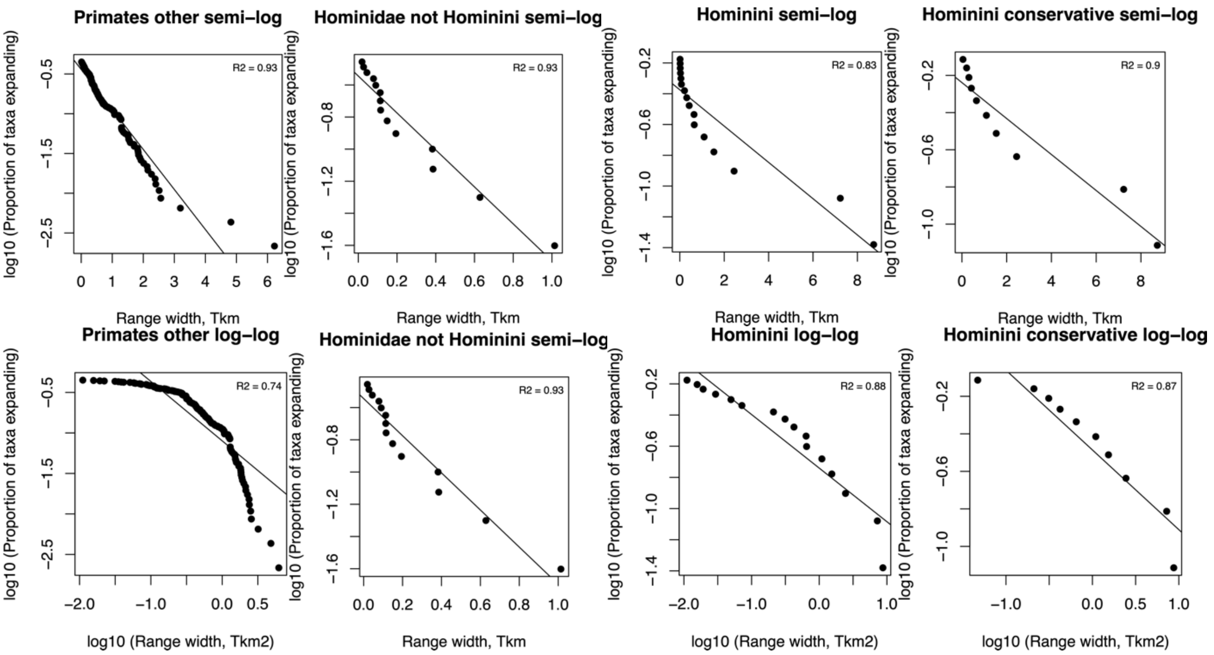

Figure S1. Alternative model fits to the global fossil record throughout the Cenozoic: (a) species survivorship, (b) range expansion.

Is the scaling of Hominini special?

We see that hominins scale in special ways as compared to the rest of the mammals analyzed. The intriguing aspect about the scaling of survivorship and expansion of Hominin is that the difference is not due to outlier points, the whole scaling relationships are different. An immediate thought would be that this scaling is due to hominins being exceptionally large or dominant. This is not the case. In fact, special scaling patterns hold even if several of the largest hominin species, including *H. sapiens*, are dropped out from the analysis. Moreover, the durations of hominin species are not exceptionally long, quite the opposite. Yet they show special scaling as well.

The best fit for species survivorship within Hominini is the pattern of Type I. This holds for the inclusive and conservative taxonomic treatments of hominins. Even in the conservative taxonomic treatment, where the difference between the statistics for Type I and Type II is small, we can see from the plots in Figure S1 that Type I (plain) has a better fit at short durations. This reassuringly suggests that the special scaling patterns for Hominini is not due to large outliers.

The best fit for range expansion within Hominini is the pattern of Type III when using the inclusive taxonomic list. When using the conservative taxonomic list, the ordinary scaling of Type II shows the best fit for range expansion. From Figure S1 we can see that conceptually Type III fit (log-log) looks more convincing in the middle of the range distribution.

We can further analyze robustness of the scaling patterns via bootstrapping, where we resample with replacement observations of occurrence of fossil taxa. We then can compute durations and ranges on bootstrapped data. Ranges and durations will not change due to duplications during bootstrapping, they will only change due to omissions. Thus, bootstrapping in our case is equivalent to random subsampling with the expectation of about 63% of the original occurrences to appear in each randomized iteration.

We run 100 randomized replicates of this experiment. Table S3 presents the statistics of prevailing scaling relationships across bootstrap iterations for each taxonomic group. The results shows that scaling patterns for Primates outside hominins are very robust. Scaling within hominins is more variable but the dominant patterns are consistent with the non-bootstrapped analysis and with the special patterns for durations standing out even stronger.

Overall, we see that the scaling patterns for hominins are special, but to what extent this may be due to overenthusiastic taxonomic splitting of hominin species in the research community remains an open question. Efforts to resolve hominin taxonomy are ongoing for many years and there is no easy solution (3). The conservative list of Martin et al. is based on macroevolutionary expectations about coexistence of lineages in time and space. Those expectations come from patterns observed across other lineages. It is, thus, not very surprising that the conservative treatment gives patterns that are closer to the ordinary scaling across other mammalian orders.

Certainly, the datasets for Hominini are small and uncertain, the model fits for this group are not very robust yet rather consistent across different analytical exercises. If the special scaling patterns for hominins truly hold in reality, this would have interesting implications for early human evolution research and beyond.

The main implication of this scaling would suggest that survivorship and expansion of hominin species is not memoryless, as for other taxonomic groups. This would imply different laws of competition for hominins rather than the classical Red Queen's competition.

The scaling of range expansion as Type III would imply that the probability of expanding increases with the range size occupied. This scaling is commonly known as “rich gets richer”. At the same time, the scaling of durations as Type I would imply aging of species. Hominin species that have existed for a longer time would have a higher probability to go extinct. In combination, these two scaling patterns would suggest competitive conditions under which one either expands to occupy large territories or perishes. Pending further investigation, this pattern could offer insights into fundamental questions of early human evolution, including potential insights into how come there is only one surviving species of humans.

Table S3. Bootstrapping analysis of Primate data. The numbers indicate how many times a particular type of scaling showed the best fit within each taxonomic group. This analysis is over 100 randomized bootstrapping replicates. Fractional numbers are due to occasional equal fitness scores for several types of scaling. Bold face indicates the prevailing scaling type for each taxonomic group.

	Counts of best fits			
	Type I (plain) Aging	Type II (semi-log) Red Queen	Type II for area	Type III (log-log) Rich gets richer
**(a) Species survivorship**				
Primates all	0	**100**	-	0
Primates not Hominidae	0	**100**	-	0
Hominidae not Hominini	7	**86**	-	6
Hominini inclusive	**74**	26	-	0
Hominini conservative	**82.5**	16.5	-	1
**(b) Species range width**				
Primates all	0	**100**	0	0
Primates not Hominidae	0	**100**	0	0
Hominidae not Hominini	0	**68.5**	3	21.5
Hominini inclusive	0	48.5	0	**51.5**
Hominini conservative	0	**82**	0	16

The counts for Hominini b) do not sum to 100 because in one case of randomized subsampling the number of points for curve fitting fell below the minimum threshold of the required number of data points.

Approximating maximum ranges of taxa as rectangular

We approximate the maximum range of a taxon as a rectangular bounding box resting on the minimum and the maximum latitude and longitude of the localities at which the taxon occurs. as shown in Figure S2. The range defined this way does not have to be occupied at the same time, although in many cases it is. This treatment allows us not to force localities into time bins, since we aim at analyzing patterns in space separately from analyzing patterns in time. We acknowledge that ranges in reality are rarely rectangular and they may be discontinuous in space, for instance, they may include water. A rectangular shape represents the upper bound of the range and works well for robustness and consistency of treatment. We include a complementary analysis with ellipses, convex hulls and geospheres in the supplement. The resulting patterns are similar and the main conclusions hold under all the variants.

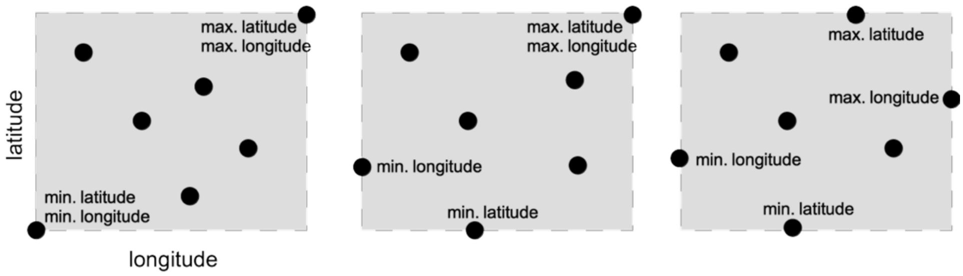

Figure S2. Estimating maximum ranges of taxa from fossil data. The left plot shows the case of two anchor localities, the middle point shows the case of three anchor localities and the right plot shows the case of four anchor localities.

Scaling of range expansion using paleocoordinates

Our analysis reported in the main text uses present day coordinates for localities. Using present day coordinates, even approximate, is our preferred design choice for this analysis of macroevolutionary laws of expansion for two reasons.

The first reason is that our focus is on the competitive expansion within the Red Queen's framework. When a tectonic plate moves, this shows a nominal expansion of the range without the actual competitive expansion. Using paleocoordinates would overestimate competitive expansion; however, since plates move relatively slowly as compared to the typical durations of taxa, we do not expect this overestimation to affect the results in a substantial way. Using present day coordinates is not ideal either, since present day coordinates can overestimate or underestimate actual ranges if individual ranges happen to lie over tectonic faults.

The second reason to treat paleocoordinates with caution is that reconstructions of paleocoordinates are at coarse scales. There are no consensus for paleocoordinates, as Figure S2 illustrates with the position of Berlin reconstructed to 30 Ma via eight tectonic models available via GPlates (4). We see from the figure that the position of Berlin varies from model to model, sometimes to an extent that the distance between the position predicted by two different models is larger than the distance from the original present-day position to either of the modelled positions. The uncertainty of using paleocoordinates is thus about as high as using present day coordinates. In addition, paleocoordinates are only available at 1 Ma intervals, which introduces extra uncertainty for species with multiple observations over short durations.

All issues considered, we do our main analysis using the coordinates of fossil localities at present day. As a sanity check here we report supplemenbtary results using paleocoordinates from two resent tectonic models: Merdith et al. 2021 (5) and Muller et al. 2022 (6). The outcomes of scaling are very similar to those using the present-day coordinates. The dominant fits change in a few cases in orders with small sample sizes. The changes as compared to the main results are indicated by arrows. The main conclusions of this study firmly hold under the analysis using paleocoordinates.

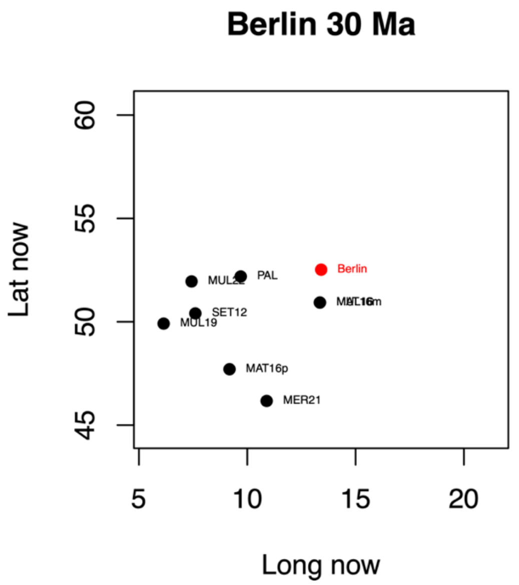

Figure S2. The geographic position of Berlin today and 30 Ma ago as predicted by eight tectonic models available via GPlates (4) : SET12 stands for the model by Seton et al 2012 (7); MER21 – Merdith et al. 2021 (5); MUL22 – Muller et al. 2022 (6); MUL19 – Muller et al. 2019 (8); MAT16m and MAT16p – Matthews et al. 2016 (9), where m stands for mantle reference and p stands for paleomag reference; PAL - Scotese et al. 2016 (10) PALEOMAP reconstructions.

Table S4. The scaling of taxon expansion throughout the Cenozoic using paleocoordinates of (6) and (5). "LAW" indicates that Type II scaling is best supported, "Lin" indicates that Type I scaling is best supported, "Pow" indicates that Type III scaling is best supported. "2" indicates that the best fit is on quadratic transformation (area rather than range width). Parentheses indicate that the best supported scaling is statistically weak, $R^2<0.9$. The orders are sorted from the most abundant in the fossil record down to less abundant. Dashes indicate no or not enough data. Arrows indicate taxonomic orders where the best fit or its statistical strength changes from the main analysis using current coordinates to using paeocoordinates.

	Patterns of range expansion of fossil taxa			
	With paleocoordinates Meridith et al. 2021		With paleocoordinates Muller et al. 2022	
	by genera	by species	by genera	by species
Rodentia	LAW	LAW	LAW	LAW
Artiodactyla	LAW	LAW	LAW	LAW
Carnivora	LAW	LAW	LAW	LAW
Perissodactyla	LAW	LAW	LAW	LAW
Eulipotyphla	LAW	LAW	LAW	LAW
Primates	LAW	(LAW)	LAW	(LAW)
Proboscidea	LAW2	LAW	LAW2	LAW
Lagomorpha	LAW	LAW	LAW	LAW
Condylarthra	(LAW) → LAW	LAW2 → LAW	(LAW) → LAW	LAW2
Chiroptera	LAW	LAW	LAW	LAW
Cimolesta	LAW	LAW	LAW	LAW
Multituberculata	LAW	Lin	LAW → Lin	Lin
Creodonta	LAW	LAW	LAW	LAW
Didelphimorphia	LAW	LAW2 → LAW	LAW	LAW2
Cingulata	LAW2 → LAW	LAW2	LAW2 → LAW	LAW2
Pilosa	(LAW) → LAW	LAW	(LAW) → Lin-	LAW
Mesonychia	Lin → LAW2	LAW	Lin	LAW
Notoungulata	LAW → LIN	-	LAW	-
Hyracoidea	Lin	Lin → (Lin)	Lin	Lin
Leptictida	-	Lin	-	Lin
Sparassodonta	LAW	-	LAW	-

Analysis with alternative shapes of ranges: ellipses, convex hulls, geosphere

Our analysis reported in the main text treats fossil species ranges as rectangular. Here, we present supplementary results with fossil ranges treated as ellipses, convex hulls and geosphere.

Ellipses: the area of an assumed elliptic range is computed as $\pi d_1 d_2/4$, where d_1 and d_2 are the distances between the maximum and the minimum latitude, and the maximum and the minimum longitude respectively. These distances are the same as for rectangulars. Here we assume that each longitudinal and latitudinal degree is 111 km. We compute the range width as the square root of the range area.

Convex Hulls: we first filter coordinates of all occurrences of a taxon to form a convex hull. We then compute the area of the convex hull in degrees. We use the library `splancs` in **R** for this. Then we convert the area into km^2 assuming that each square degree is equal to $(111\ km)^2$. We compute the range width as the square root of the range area.

Geosphere: here we use the coordinates for convex hulls obtained using the same procedure as above. We then compute the area in km^2 of within that convex hull, assuming a geosphere model. We do this using the package `geosphere` in **R**. We compute the range width as the square root of the range area.

Within the four treatments of fossil ranges (rectangular, ellipse, convex hull and geosphere) the rectangular would offer the upper bound of the range, followed by the ellipse, the convex hull and the geosphere in the descending order of range area estimates for the same set of coordinates.

Figure S3 illustrates the relationships between these treatments. Table S5 shows the results of scaling of taxon ranges using ellipses, convex hulls and the geosphere. Arrows indicate changes of best fit relationships or their statistical strength. We see that the results for the largest orders firmly hold, changes happen mainly in the geosphere treatment and primarily concern the statistical strength of relationships within the orders that have small number of observations. Our main conclusions hold under either of the four treatments of range shapes.

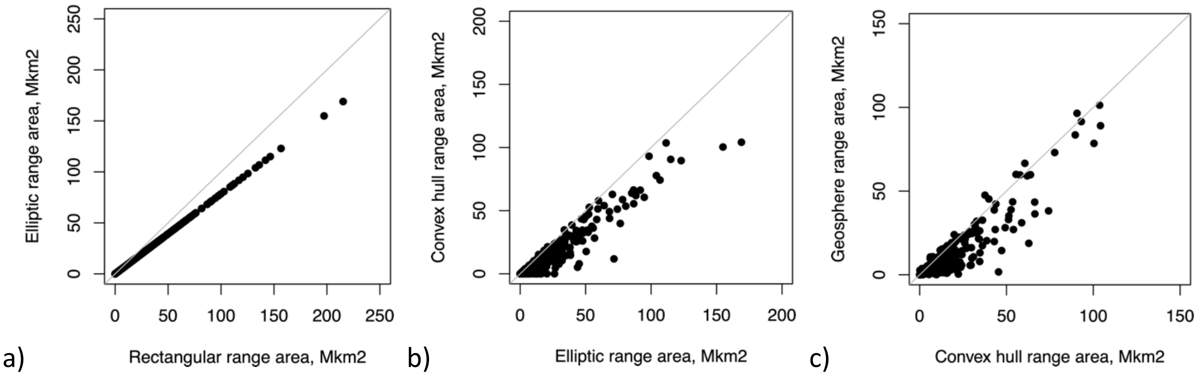

Figure S3. Correspondence between ranges estimates using different base shapes: a) rectangular vs. elliptic, b) elliptic vs. convex hull, c) convex hull vs. geosphere.

Table S5. The scaling of taxon expansion throughout the Cenozoic using different shapes for range area estimates. “LAW” indicates that Type II scaling is best supported, “Lin” indicates that Type I scaling is best supported, “Pow” indicates that Type III scaling is best supported. “2” indicates that the best fit is on quadratic transformation (area rather than range width). Parentheses indicate that the best supported scaling is statistically weak, R^2<0.9. The orders are sorted from the most abundant in the fossil record down to less abundant. Dashes indicate no or not enough data. Arrows indicate taxonomic orders where the best fit or its statistical strength changes as compared to the main analysis using rectangular ranges.

	Patterns of range expansion of fossil taxa					
	Ranges as ellipses		Ranges as convex hulls		Ranges as convex hulls, geosphere	
	by genera	by species	by genera	by species	by genera	by species
Rodentia	LAW	LAW	LAW	LAW	LAW	LAW
Artiodactyla	LAW	LAW	LAW	LAW	LAW	LAW
Carnivora	LAW	LAW	LAW	LAW	LAW	LAW
Perissodactyla	LAW	LAW	LAW	LAW	LAW	LAW
Eulipotyphla	LAW	LAW	LAW	LAW	LAW	LAW
Primates	LAW	(LAW)	LAW	(LAW)	LAW	(LAW) → (Pow)
Proboscidea	LAW2	LAW	LAW2	LAW	LAW2 → Lin	LAW
Lagomorpha	LAW	LAW	LAW	LAW	LAW	LAW
Condylarthra	LAW	LAW2	(LAW)	LAW2	(LAW) → LAW	LAW2
Chiroptera	LAW	LAW	LAW	LAW	LAW → (LAW)	LAW → (LAW)
Cimolesta	LAW	LAW	LAW	LAW	LAW	LAW
Multituberculata	LAW	Lin	LAW → LAW2	Lin → LAW2	LAW → LAW2	Lin → LAW2
Creodonta	LAW	LAW	LAW	LAW	LAW	LAW
Didelphimorphia	LAW		-	LAW2 → Lin	-	LAW2 → ~Lin
Cingulata	LAW2	LAW2	-	-	LAW2 → LAW	-
Pilosa	LAW	LAW	-	-	-	-
Mesonychia	Lin	LAW	Lin	-	Lin	-
Notoungulata	LAW		-	-	-	-
Hyracoidea	Lin	Lin	Lin	-	Lin	-
Leptictida		Lin	-	-	-	-
Sparassodonta	LAW		-	-	-	-

References (Supplement)

1. Martin JM, Leece AB, Baker SE, Herries AIR, Strait DS. A lineage perspective on hominin taxonomy and evolution. Evol Anthropol Issues News Rev. n/a(n/a):e22018.

2. Van Valen L. A new evolutionary law. Evol Theory. 1973;1:1–30.

3. van Holstein LA, Foley RA. A process-based approach to hominin taxonomy provides new perspectives on hominin speciation. Evol Anthropol Issues News Rev. 2022;31(4):166–74.

4. Kocsis AT, Raja NB, Williams S, Forschungsgemeinschaft D, Nordbayern FG. rgplates: R Interface for the GPlates Web Service and Desktop Application. 2023.

5. Merdith AS, Williams SE, Collins AS, Tetley MG, Mulder JA, Blades ML, et al. Extending full-plate tectonic models into deep time: Linking the Neoproterozoic and the Phanerozoic. Earth-Sci Rev. 2021 Mar 1;214:103477.

6. Müller RD, Flament N, Cannon J, Tetley MG, Williams SE, Cao X, et al. A tectonic-rules-based mantle reference frame since 1 billion years ago – implications for supercontinent cycles and plate–mantle system evolution. Solid Earth. 2022 Jul 7;13(7):1127–59.

7. Seton M, Müller RD, Zahirovic S, Gaina C, Torsvik T, Shephard G, et al. Global continental and ocean basin reconstructions since 200Ma. Earth-Sci Rev. 2012 Jul 1;113(3):212–70.

8. Müller RD, Zahirovic S, Williams SE, Cannon J, Seton M, Bower DJ, et al. A Global Plate Model Including Lithospheric Deformation Along Major Rifts and Orogens Since the Triassic. Tectonics. 2019;38(6):1884–907.

9. Matthews KJ, Maloney KT, Zahirovic S, Williams SE, Seton M, Müller RD. Global plate boundary evolution and kinematics since the late Paleozoic. Glob Planet Change. 2016 Nov 1;146:226–50.

10. Scotese C. PALEOMAP PaleoAtlas for GPlates and the PaleoData Plotter Program, PALEOMAP Project. 2016; Available from: http://www.earthbyte.org/paleomappaleoatlas-for-gplates/
